# 正常核型成人急性髓系白血病的基因突变谱及其临床特征分析

**DOI:** 10.3760/cma.j.issn.0253-2727.2021.05.012

**Published:** 2021-05

**Authors:** 爱杰 黄, 磊 高, 雄 倪, 晓霞 胡, 古生 唐, 辉 程, 洁 陈, 莉 陈, 丽霞 刘, 程程 王, 卫平 章, 建民 杨, 健民 王

**Affiliations:** 1 海军军医大学第一附属医院（长海医院）血液科，中国人民解放军血液病研究所，上海 200433 Department of Hematology, Institute of Hematology, the First Affiliated Hospital of Navy Military Medical University (Changhai Hospital), Shanghai 200433; 2 北京金橡生物科技有限公司 100176 Acornmed Biotechnology Co., Ltd. Beijing, 100176

急性髓系白血病（AML）是以髓系原始细胞克隆性增殖及分化受阻，抑制正常造血为特征的异质性疾病[1]–[2]。40％～50％的AML患者为正常核型（normal karyotype，NK）[1],[3]–[5]，此类患者预后同样具有异质性，伴有众多的基因突变，可能影响化疗方案的选择[3]。应用靶向基因测序技术有助于检出多重体细胞突变基因。近年研究发现PLT与白血病细胞的恶性增殖及干性维持密切相关，我们的前期研究结果提示，AML初诊时外周血PLT较高可能与不良预后相关[6]–[7]，其他学者亦有类似报道[8]–[9]。本研究中我们对134例NK-AML初发标本进行基因突变检测，以探究其基因谱系改变及初发时PLT特征。

## 病例与方法

1. 研究对象：2010年1月至2019年1月共265例AML患者在我院初诊及治疗，将初诊时染色体核型正常的134例（50.6％）患者纳入本研究。按欧洲白血病网络（ELN）2017标准进行危险分层，其中低危组32例，中危组68例，高危组34例。所有患者初诊时均进行了骨髓细胞形态学、免疫学、细胞遗传学及分子生物学的检测，疾病诊断参照WHO 2016版分类标准[10]。初诊时中位年龄为49（11～75）岁，其中男70例（52.2％），初诊外周血中位WBC、HGB及PLT分别为9.75（0.54～276.30）×10^9^/L、92（34～148）g/L、67.5（3.5～392.0）×10^9^/L，骨髓中位原始细胞比例为68.5％（21％～98％）。89例患者仅接受化疗，45例接受异基因造血干细胞移植（allo-HSCT）。所有患者均留有初诊时的DNA样本，研究方案由本单位伦理委员会批准，研究过程符合赫尔辛基宣言，所有研究对象均签署知情同意书。

2. 染色体检测：初诊时留取骨髓液，用直接和短程培养法培养（24～48 h），在收集骨髓细胞前1 h加秋水仙碱，制备骨髓细胞染色体标本，进行R显带，并根据人类细胞遗传学命名法（ISCN 2009）进行核型的描述和记录[11]–[13]。

3. 测序方法：将初诊时的骨髓分离单个核细胞后提取DNA，并保存于−20°C。利用文库构建试剂盒（美国KAPA Biosystems公司产品）制备全基因组文库，采用髓系肿瘤相关的210个基因板对基因组文库目标区进行捕获、定量。使用Illumina Novaseq测序平台测序。采用下列标准筛选原始变异结果：每个样本的平均有效测序深度≥1000×；对于单个核变异、插入或删除，等位基因突变频率≥0.5％；读取片段均通过高质量评估；突变基因在阴性及阳性组中分别进行确认[14]。通过检索dbSNP、1000genomes、EXAC数据库，定义健康人群中突变频率>1％的位点为基因多态性位点，在分析时排除该部分数据。

4. 治疗方案：诱导化疗方案参见我们既往报道[15]。诱导治疗后达完全缓解（CR）的患者接受4个疗程巩固化疗（阿糖胞苷2 g/m^2^每12 h 1次，第1～3天），如有合适供者，巩固治疗2～4个疗程后行allo-HSCT。移植患者预处理方案参见既往报道[15]–[16]。接受无关或半相合供者allo-HSCT的患者需使用抗胸腺细胞球蛋白，所有患者在移植后均接受环孢素、短程甲氨蝶呤及霉酚酸酯预防移植物抗宿主病。

5. 疗效及预后标准：血液学CR定义为骨髓中原始细胞比例<5％，无髓外侵犯，外周血细胞恢复（ANC>1.0×10^9^/L，PLT>100×10^9^/L，不依赖红细胞输注）。当骨髓中原始细胞比例≥5％或有髓外白血病表现时定义为疾病复发。总生存（OS）时间定义为自诊断日期至患者死亡或随访截止日期。无复发生存（RFS）时间定义为患者达血液学CR日期至患者死亡、第一次复发或随访截止日期。

6. 随访：采用门诊、住院电子病案查阅或电话随访。随访截止时间为2019年5月31日，中位随访时间为49.5（0.8～114.3）个月。

7. 统计学处理：统计分析利用SPSS 18.0、R 3.6.2软件完成，生存分析（OS及RFS）利用Kaplan-Meier法，采用Log-rank检验比较组间生存率差异。诊断至移植的中位时间为175 d，缓解至移植的中位时间为119 d，以此为分界点，采用Landmark法分析移植对预后的影响。采用卡方检验及Fisher精确检验进行组间比较。累积复发率（CIR）及无复发死亡率（NRM）采用竞争风险模型分析及Gray's检验。单因素及多因素分析采用Cox比例风险回归分析，单因素分析中*P*<0.1的变量纳入多因素分析，其中治疗方式（化疗或移植）以时依协变量纳入分析。*P*<0.05为差异有统计学意义。

## 结果

1. 多基因突变谱及共存互斥分析：134例患者中有133例至少检出1种基因突变，中位基因突变数为6（0～13）种，共涉及128种基因，其中共25种基因在≥10例患者中检出（图1）。突变率较高的前五位基因分别为NPM1、CEBPA、TET2、DNMT3A、NRAS（均>20％）。中位等位基因变异频率（VAF）为0.4387（0.005～1），其中信号通路相关基因突变负荷较低，提示这类基因倾向于在疾病晚期发生突变。

**图1:**
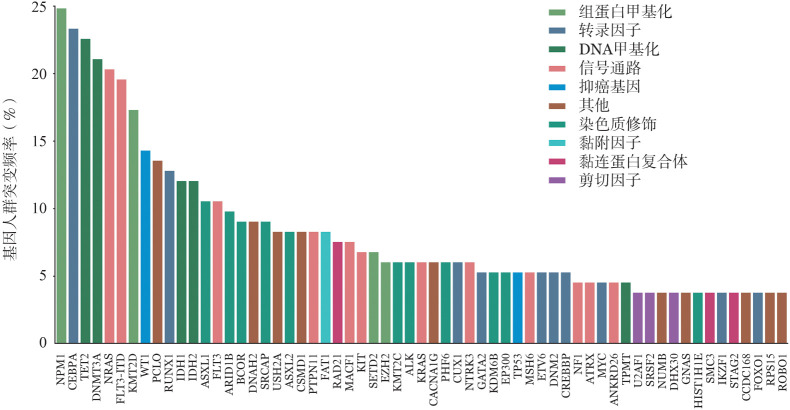
134例正常核型急性髓系白血病患者多基因突变图谱

突变基因之间存在广泛共存及互斥情况（图2），提示NK-AML患者中的遗传复杂及不稳定性。共存的突变基因有NPM1与DNMT3A、NPM1与FLT3-ITD、CSMD1与IDH2、TET2与KMT2D、CEBPA与FAT1、DNAH2与FLT3、NPM1与PTPN11、IDH1与DNMT3A、BCOR与RUNX1（*P*值均<0.05）。而突变基因IDH1与TET2、CEBPA与FLT3-ITD、FLT3与CEBPA、IDH2与NRAS、WT1与NPM1、WT1与KMT2D、PCLO与NPM1之间是互斥的（*P*值均<0.05）。突变基因中位突变位点数为7（0～18）个，大多为错义突变，不同基因突变模式存在差异。单个患者检出突变类型多样，最主要的仍为错义突变。

**图2:**
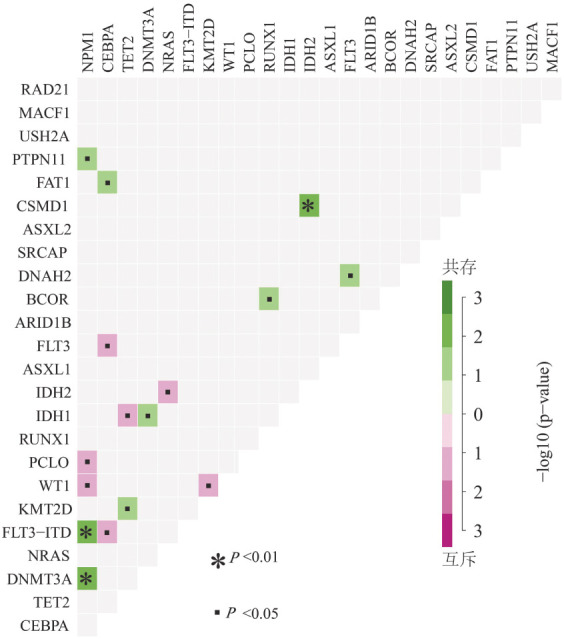
突变基因共存互斥关联分析（≥10例患者中检出）

2. 突变基因功能分析：将突变基因按照功能进行聚类分析，常见突变基因涉及的通路有表观遗传学（28/128，21.9％）、信号通路（35/128，27.3％）、转录因子（17/128，13.3％）、剪切因子（7/128，5.5％）、黏连蛋白复合体（5/128，3.9％）、抑癌基因（6/128，4.7％）、细胞代谢（1/128，0.8％）、其他（29/128，22.6％）。信号通路相关基因最为常见，其中>5例患者检出的基因分别为NRAS、FLT3-ITD、FLT3、PTPN11、MACF1、KIT、KRAS、NTRK3、MSH6、NF1、ATRX、ANKRD26。

表观遗传学相关的基因突变较常见，其中包括DNA甲基化（TET2、DNMT3A、IDH1、IDH2、TPMT，5/128，3.9％）、组蛋白甲基化（NPM1、KMT2D、SETD2、EZH2、NSD2、KMT2A，6/128，4.7％）、染色质修饰（ASXL1、ARID1B、BCOR、SRCAP、ASXL2、KMT2C、ALK、PHF6、EP300、KDM6B、HIST1H1E、KDM6A、BLM、BCORL1、SETBP1、SUZ12、TRIM24，17/128，13.3％）。

3. 携带不同突变基因NK-AML初诊时PLT特征：对伴突变频率前十位基因突变患者进行初诊时PLT特征分析，结果显示CEBPA双突变与PLT呈负相关（*r*＝−2.03，*P*＝0.019），CEBPA双突变较单突变或无突变患者初诊时PLT明显减低［（47.9±70.3）×10^9^/L对（86.3±70.5）×10^9^/L，*P*＝0.019）］。NPM1^+^FLT3-ITD^−^及DNMT3A基因突变者初诊时PLT高于无突变者［NPM1^+^FLT3-ITD^−^：（104.4±97.9）×10^9^/L对（68.0±61.1）×10^9^/L，*P*＝0.036；DNMT3A：（119.3±84.6）×10^9^/L对（69.3±64.4）×10^9^/L，*P*＝0.001］。

4. 基因突变对预后的影响：如表1所示，将患者的临床特征与突变频率前十位突变基因进行关于生存的单因素分析，结果显示，两个疗程未达CR、第2个疗程后流式细胞术检测的微小残留病（FCM-MRD）及巩固治疗前FCM-MRD≥0.1％、基因突变数目≥7、剪切因子基因突变、信号通路相关基因突变、DNMT3A突变者OS及RFS均较差（*P*值均<0.05），年龄>49岁、仅接受化疗对患者RFS有影响，而初诊时WBC>10×10^9^/L对OS有影响。多因素分析结果显示仅接受化疗、初诊时WBC>10×10^9^/L、基因突变数目≥7、剪切因子基因突变、DNMT3A基因突变与较差的OS相关。仅接受化疗、巩固治疗前FCM-MRD≥0.1％、基因突变数目≥7、剪切因子相关基因突变、DNMT3A基因突变的患者更易复发，为RFS的独立不良预后因素。

**表1:** 134例正常核型急性髓系白血病患者生存影响因素的单因素及多因素分析

临床特征	OS				RFS			
	单因素		多因素		单因素		多因素	
	*HR*（95％*CI*）	*P*值	*HR*（95％*CI*）	*P*值	*HR*（95％*CI*）	*P*值	*HR*（95％*CI*）	*P*值
年龄（>49岁对≤49岁）	1.496（0.929~2.409）	0.098	1.052（0.598~1.852）	0.860	1.623（1.038~2.539）	0.034	1.198（0.687~2.089）	0.523
性别（男对女）	1.133（0.705~1.820）	0.607	–	–	0.917（0.589~1.427）	0.700	–	–
治疗方式（化疗对移植）	5.061（0.758~33.786）	0.094	16.589（1.528~180.111）	0.021	7.603（1.533~37.718）	0.013	10.158（1.752~58.888）	0.010
WBC（>10×10^9^/L对≤10×10^9^/L）	1.699（1.053~2.743）	0.030	2.985（1.591~5.597）	0.001	1.425（0.914~2.222）	0.118	–	–
PLT（>40×10^9^/L对≤40×10^9^/L）	1.156（0.703~1.900）	0.568	–	–	0.958（0.607~1.511）	0.852	–	–
HGB（>92g/L对≤92g/L）	0.980（0.609~1.578）	0.935	–	–	0.997（0.636~1.560）	0.988	–	–
LDH（>252U/L对≤252U/L）	1.134（0.707~1.818）	0.603	–	–	0.867（0.557~1.352）	0.530	–	–
初发骨髓原始细胞（>68％对≤68％）	1.144（0.712~1.836）	0.579	–	–	0.953（0.613~1.481）	0.829	–	–
ECOG评分								
1对 0	0.586（0.206~1.669）	0.317	–	–	0.645（0.228~1.821）	0.407	–	–
2对 0	0.957（0.337~2.717）	0.935	–	–	1.140（0.405~3.202）	0.804	–	–
3对 0	1.275（0.341~4.772）	0.718	–	–	1.360（0.363~5.097）	0.648	–	–
两个疗程内达CR（否对是）	1.982（1.228~3.200）	0.005	1.455（0.827~2.565）	0.194	1.691（1.084~2.636）	0.020	1.268（0.697~2.305）	0.436
FCM-MRD（≥0.1％对<0.1％）								
第1个疗程后	1.363（0.836~2.223）	0.215	–	–	1.447（0.918~2.280）	0.112	–	–
第2个疗程后	1.813（1.105~2.974）	0.019	1.230（0.610~2.479）	0.563	1.979（1.243~3.149）	0.004	1.262（0.666~2.389）	0.476
巩固治疗前	1.983（1.156~3.401）	0.013	1.297（0.624~2.695）	0.487	2.321（1.404~3.838）	0.001	2.220（1.209~4.075）	0.010
基因突变数目（≥7对<7）	2.188（1.344~3.562）	0.002	1.912（1.031~3.546）	0.040	1.927（1.230~3.018）	0.004	2.248（1.233~4.101）	0.008
基因涉及通路（有对无）								
剪切因子	2.157（1.250~3.721）	0.006	3.453（1.747~6.825）	<0.001	2.006（1.177~3.421）	0.011	2.146（1.080~4.265）	0.029
信号通路	2.744（1.398~5.386）	0.003	2.276（0.961~5.387）	0.061	2.394（1.315~4.361）	0.004	1.602（0.760~3.377）	0.216
DNA甲基化	1.115（0.688~1.806）	0.659	–	–	1.217（0.774~1.914）	0.394	–	–
DNMT3A（突变对无突变）	1.917（1.113~3.302）	0.019	1.949（1.012~3.754）	0.046	1.772（1.071~2.931）	0.026	2.076（1.111~3.880）	0.022

注：OS：总生存；RFS：无复发生存；ECOG：美国东部肿瘤合作组；CR：完全缓解；FCM-MRD：流式细胞术检测的微小残留病

基因突变数目≥7、发生剪切因子基因突变、DNMT3A突变的患者3年OS率及3年RFS率较无突变的患者低［基因突变数目≥7：OS：（31.4±6.4）％对（59.7±6.3）％，*P*＝0.001，RFS：（25.6±5.9）％对（49.6±6.4）％，*P*＝0.003；剪切因子基因突变：OS：（19.0±9.1）％对（53.4±5.1）％，*P*＝0.005，RFS：（21.1±8.8）％对（42.2±5.0）％，*P*＝0.008；DNMT3A：OS：（30.7±9.7）％对（51.1±5.2）％，*P*＝0.017，RFS：（16.9±7.6）％对（43.8±5.1）％，*P*＝0.023］。Landmark分界点后生存结果显示：allo-HSCT可改善DNMT3A突变患者OS及RFS［移植（12例）对化疗（16例），18个月OS率：（57.7±16.3）％对（18.8±15.5）％，*P*＝0.110；18个月RFS率：（45.8±16.7）％对（12.5±11.7）％，*P*＝0.025］，并可降低该类突变患者的复发率［18个月CIR：（12.5±1.6）％对（53.1±5.3）％，*P*＝0.031］，但对基因突变数目≥7、剪切因子基因突变患者的生存无明显影响（OS、RFS、CIR及NRM：*P*值均>0.05）。

## 讨论

NK-AML患者常伴有一系列的基因突变，如NPM1、FLT3-ITD、CEBPA、DNMT3A、IDH1/2D突变[5]。近年来，二代测序（NGS）技术已广泛应用于AML疾病的诊断、预后及MRD的监测[17]–[19]。本研究意在分析除外核型影响因素下，NGS结果对NK-AML患者的分层及预后价值。在本研究中99.3％（133/134）的NK-AML患者检出了至少一种突变，≥10例患者中检出的突变基因共26个。按ELN2017标准，本研究中134例NK-AML患者低危、中危、高危组分别占23.9％、50.7％、25.4％，提示NK-AML有较大的异质性。

本研究多因素分析显示高基因突变数目（≥7）、剪切因子基因突变、DNMT3A基因突变为生存的独立危险因素，与既往文献报道相似[20]–[26]。ELN 2017诊断标准中未纳入DNMT3A突变，中国AML2017版诊疗指南将DNMT3A突变、剪切因子基因突变放至次级检查中，在不伴有几种预后良好细胞遗传学改变时提示预后不良[27]。

本研究中DNMT3A基因突变检出率为20.9％（28/134），涉及19个突变位点，其中R882为最常见的突变位点（11/28，39.3％），其次为R326（4/28，14.3％），4例患者标本中检测出2个位点突变。50％（14/28）的DNMT3A突变患者2个疗程内达CR，余14例中2例持续NR。与齐悦等[25]报道相似，多因素分析示DNMT3A突变为RFS的独立预后危险因素，DNMT3A突变患者的生存时间明显缩短，但本研究另外发现allo-HSCT可以改善DNMT3A突变NK-AML患者的OS及RFS，且可降低复发率。

体外实验表明血小板可促进AML原始细胞增殖及细胞因子释放[28]。我们曾对174例中危组AML患者分析发现，仅接受化疗的患者中初诊时PLT>40×10^9^/L者生存较差[7]。本研究依此将患者分为高、低PLT组，生存分析提示在化疗患者中两组生存差异无统计学意义，可能与入选病例不一致，且本组病例数少相关。Thol等[29]报道DNMT3A突变在NK-AML更为多见（27.2％，71/261），突变患者起病时PLT显著高于无突变患者［中位PLT：71（9～336）×10^9^/L对44（3～624）×10^9^/L，*P*＝0.014］。DNMT3A突变患者TPO/MPL信号通路上调，同时伴有其他参与血小板活化的基因的过表达，如Pdgfb，转录组学的异常导致造血干/祖细胞的异常增殖，进而促进疾病发生[30]。本研究有类似发现，NPM1^+^FLT3-ITD^−^或DNMT3A基因突变者PLT明显增高。Thiede等[31]的研究纳入1485例AML患者，发现NPM1突变与高PLT显著相关（*P*<0.001），推测携带有NPM1突变的原始细胞保留有促进血小板分化的能力。Hsu等[32]的研究显示转染了C端缺失的NPM1突变体的K562细胞系具有更强的巨核细胞分化能力。此外，本研究显示CEBPA双突变与低PLT相关，与韩聪等[33]的报道一致。

综上所述，基因突变在NK-AML中发生比例较高，高突变基因数目（≥7）、剪接体基因突变、DNMT3A突变的患者预后差，移植可改善DNMT3A突变患者的生存。不同遗传学改变谱系的NK-AML患者初诊时中位PLT不同。对NK-AML患者行NGS检测有助于此类患者的预后分层评估及治疗选择。
